# Protocol for a study on vicarious resilience in service providers for victims and survivors of violence

**DOI:** 10.1371/journal.pone.0283474

**Published:** 2023-03-23

**Authors:** Alyssa Ferns, Benjamin S. Roebuck, Diana McGlinchey, Patricia L. Sattler, Hannah Scott, Kyle D. Killian, Theresia Bedard, Amy Boileau, Connor Tague, Katherine Thompson

**Affiliations:** 1 Victimology Research Centre, Algonquin College, Ottawa, Ontario, Canada; 2 National Policing Institute, Arlington, VA, United States of America; 3 Faculty of Social Sciences and Humanities, Ontario Tech University, Oshawa, Ontario, Canada; 4 School of Social and Behavioral Sciences, Capella University, Minneapolis, MN, United States of America; Public Library of Science, UNITED STATES

## Abstract

Few national studies examine victim service providers (VSPs), the important work that they do, and the resources and strategies contributing to their wellness at work. The proposed study aims to investigate the vicarious resilience of those working within the Canadian victim services sector. Participants will be asked about the ways in which they have changed and experienced resilience through exposure to supporting their clients, in addition to the challenges and barriers that still exist. A mixed-methods study incorporating an online survey, virtual focus groups, and semi-structured in-depth interviews will explore job satisfaction, compassion fatigue, turnover intention, instances of workplace microaggressions, vicarious resilience, coping strategies and self-care of VSP participants. The results will contribute to the literature on themes related to the wellness of VSPs. Dissemination of results will provide a Canadian perspective on organizational wellness, including challenges encountered as a result of COVID-19, working conditions that require further advocacy, and emerging perspectives on protective factors.

## Introduction

There is a growing sector of victim service providers (VSPs) tasked with supporting victims of crime from initial disclosure through to supporting victims’ families when an offender is granted parole. High-profile victims of violence and parents of murdered children who experienced further trauma through their interactions with the criminal justice system developed many of these services through lobbying efforts and advocacy [1, 2]. Statistics Canada has conducted bi-annual reports on victim services across the country. The most recent, was conducted in 2011/2012, which included responses from 760 VSPs and six criminal injuries compensation programs that reported serving 460,000 victims of crime that year [3]. Despite the rapid expansion of services for victims of crime, and the added stressors to this sector with the COVID-19 pandemic, there has been limited research on the victim services sector in Canada. Moreover, there remains a gap in the literature discussing the training needed to enhance job performance, how VSPs balance helping people with their own personal well-being, and the personal changes that can take place for those that do trauma work. Trauma work affects the people who do it in complex ways. Potential harms are well documented and described as vicarious trauma, secondary stress, compassion fatigue, or burnout [4–10]. These concepts are used to explain the possible consequences of repeated and prolonged exposure to others’ trauma and how the burden of care can manifest itself in negative physical, mental, emotional, or spiritual symptoms. Contemporary training resources in the trauma field include some discussion of the potential for vicarious trauma and the need to practice effective self-care [11–14]. Recently, scholars have critiqued the concept of vicarious trauma as one-sided, arguing that it excludes the positive effects that people may derive from trauma work, captured in the concept of vicarious resilience. That is, helping people in crisis can expose the helper to others’ positive coping skills, problem-solving, and courage, in turn building the helper’s personal capacity to respond to adversity [15–17]. This broader conceptualization is leading to innovation in research on occupational stress, as well as promising quantitative measures such as the Vicarious Resilience Scale [18].

While self-care is necessary when working in the trauma field, most resilience researchers caution that resilience is not an individual process [19] but rather an interaction between one’s personal capacity and the available, accessible resources that may be surround them [20–26]. The emerging literature on trauma-informed care emphasizes the importance of trauma-informed organizations, recognizing the vital role that organizational structures play in the well-being of clients and service providers alike [11]. Learning about the resources VSPs have found most helpful, their negotiation processes, and the obstacles they encountered enhances our understanding of vicarious resilience.

The proposed study seeks to provide an update on victim service data in Canada, while also providing a new understanding of vicarious resilience for VSPs. This project will provide an overview of the landscape of best practices across different victim service sectors, as well as locate systemic barriers that exist for VSPs. This study is especially timely given the additional stressors in the workplace as a result of or in response to the COVID-19 pandemic. Results will help to explain how VSPs can be better supported, as they continue the important work of serving clients while also maintaining their own personal wellness.

## Materials/methods

### Study design

We plan to create a multi-method study through consultation with sector professionals and the Federal-Provincial-Territorial (FPT) Working Group on Victims of Crime. We will form a methods working group and meet quarterly to offer direct feedback on the building of qualitative questions and quantitative scales, and also to pilot versions of the draft survey. Moreover, we plan to solicit further feedback from both Statistics Canada and the Department of Justice to help in finalizing the survey. Project partners and collaborators will have a direct and meaningful opportunity to shape our final research design.

The study plans to include three distinct components: 1) online survey; 2) virtual focus groups; and 3) virtual semi-structured interviews. The survey instrument explores several domains including participants’ demographics and organizational characteristics, job satisfaction, turnover intention, instances of workplace microaggressions, challenges as a result of COVID-19, a screener for Posttraumatic Stress Disorder (PTSD), compassion satisfaction, burnout and fatigue, vicarious resilience, and coping strategies. Upon completion of the survey, participants will be provided with the Project Director’s email to reach out if they would like to provide a deeper reflection on these topics via a semi-structured interview. Focus groups will be held via an online Victim Services and Vicarious Resilience research conference. This conference will also help to promote the survey to VSPs across Canada. All instruments will be translated and available in both official languages. This study was approved by the Research Ethics Board of Algonquin College (Protocol #: 2021-JUN-ROEBUCK).

### Participant sample

We will recruit a convenience sample of participants from VSPs and collaborators of the Victimology Research Centre. We will also heavily promote this study in collaboration with a national victim services and vicarious resilience research conference.

### Data collection

Our sample size justification estimates a population of 20,000 victim service providers in Canada, and so with an accepted margin of error of 5% and a confidence level of 95% we require a sample of N = 377. However, given the length of time since the Statistics Canada survey of VSPs that included 760 participants [3], we hope to be able to secure 800–1,000 participants across all provinces and territories.

Survey data will be collected using Survey Monkey. Participants must read and complete the informed consent procedures prior to beginning the survey; if they do not consent, Survey Monkey will take them to an exit screen. The survey is expected to take approximately 30 minutes to complete. Upon survey completion, participants will be offered a detailed debriefing and list of resources and mental health services (e.g., Crisis Text Line, Hope for Wellness Help Line, etc.). Data will only be included for participants who complete 80% of the survey at minimum. Survey completers will be provided information about the opportunity to participate in an in-depth interview or focus groups; instructions will be included on how to contact the Project Director via email, if interested.

Interviews will be conducted virtually via Zoom by trained members of the research team. Prior to the start of the interview, participants will be provided a link to an online consent form; a brief demographic survey will also be copied into the Zoom chat for the participant to read and complete. Interviewers will discuss any aspects of the informed consent and answer participant questions as needed before beginning the interview. Using a semi-structured interview guide, we will explore themes related to participants’ work experience and wellness. We will aim to conduct approximately 20 interviews. We will offer interview participants a $20 e-gift certificate honorarium. Interviews will be audio-recorded and transcribed verbatim.

Focus groups will occur during the Victim Services and Vicarious Resilience research conference. Interested participants will be asked to remain in the main virtual conference room following the keynote address before being randomly assigned to a virtual breakout room. The goal is to hold four concurrent focus groups with five participants in each breakout room. Upon entry into the breakout room, all participants will be provided a link to an online consent form; a brief demographic survey will be copied into the breakout room chat. Participants can read and complete the consent form individually on their own private computer. Focus group facilitators will discuss any aspects of the informed consent and answer participant questions as needed prior to the start of the focus group. Participants may consent to participate, but not consent to being recorded; these participants will be directed to an unrecorded focus group session not affiliated with the study. One researcher facilitator per focus groups session will hold a 45-minute discussion of topics related to the survey (e.g., changes experienced as a result of victim services work, organizational wellness resources available, etc.). The audio and chat files of each participating room will be recorded for coding and analysis. All study data will be confidential and will be stored in password-protected computers and all data will be accessed through password protected software.

### Measures

The survey instrument will include standardized measures, demographic and organizational characteristics, as well as open-ended questions (Table 1).

**Table 1:** Table of measures.

Measure	Items
**Participant Demographics** (Item developed for this study)	Data collected on service providers’ and volunteers’ age, gender identity, sexual identity, relationship status, language, ethnic and cultural origins, religion or spirituality, education, field of study, and social service accreditation.
**Organizational Information** (Item developed for this study)	Data collected on service providers’ and volunteers’ current employment, type of organization, organization mandate, number of staff members, and number of volunteers.
**Employment/Volunteer Information** (Item developed for this study)	Data collected on location of employment and volunteer work, position title and role, type and length of position, union status, salary, and COVID-19 considerations.
**Job Satisfaction Facet Item** (PAJS-FI; 2018)	An 11-item measure that assesses level of satisfaction with various aspects of the service provider’s job.
**Turnover Intention** (2015) (Modified)	A 2-item measure that assesses intent to leave the organization. Items include ideations about quitting and plans to search for a new job in the next 12 months.
**Organization Structure and Wellness** (Open-ended question)	How does your organizational structure affect your well-being? This could include your work environment, compensation, benefits, flexibility, supervision model, or other aspects of your work life.
**COVID-19** (Item developed for this study)	Changes to workload, number of clients, level of stress, work-life balance, overall mental health.
**Microaggressions in the workplace** (Item developed for this study)	Assesses intentional and unintentional verbal, behavioral, or environmental indignities in the workplace. Assesses whether participants have witnessed or experienced microaggressions.
**Workplace safety** (Item developed for this study)	Service provider experiences of physical or sexual violence, harassment or threatening behaviour, or workplace bullying
**Primary Care PTSD Screen for DSM-5** (PC-PTSD-5; 2015)	A 5-item measure that identifies respondents with probable PTSD as a result of unusually or especially frightening, horrible, or traumatic events.
**The Professional Quality of Life Scale** (ProQOL Version 5; 2009)	A 30-item self-report measure of the negative and positive effects of helping others who experience suffering and trauma. The ProQOL has subscales for compassion satisfaction, burnout, and compassion fatigue.
**Vicarious Resilience Scale** (VRS; 2017)	A 27-item measure that assesses service providers’ and volunteers’ experiences of vicarious resilience through trauma work. Assesses changes in how a professional views their clients, their approach to this work, and/or their worldview since beginning trauma work.
**Coping Strategies Inventory** (2019)	A 20-item measure that assesses service provider and volunteer engagement in coping strategies and activities.
**Self-Care** (Open-ended question)	How do you care for yourself while doing trauma work? What have you found most helpful?
**Vicarious Posttraumatic Change** (Open-ended question)	How has working or volunteering with victims or survivors of crime affected you personally? Have you changed? What have you learned?
**COVID-19 and final thoughts** (Open-ended question)	Is there anything else you would like to share with us? For example, how you have been affected by the COVID-19 pandemic, thoughts about the survey content, or other things we might have missed?

#### Participants’ demographics and organizational characteristics

In collaboration with sector professionals and previous research, we will finalize demographic questions and questions about participants’ organizational characteristics. Demographic questions will include age, ethnic origin, gender and sexual identity, relationship status, number of dependents in the household, religious affiliation, highest level of education, field of study, and professional accreditations. Participants’ organizational characteristics will include the province/territory, organization type (e.g., government vs. non-government), organization description (e.g., urban vs. rural), number of staff, organizational mandate(s) for specifics groups and particular crime types, position title, length of tenure, description of role (e.g., frontline vs. indirect), approximate number of clients served weekly, and a description of the physical environment pre-pandemic as well as any changes that occurred during the pandemic. We will also ask participants to identify the 1) reasons for choosing their field; 2) feelings of preparedness for their position; 2) managerial responsibilities, if any; 3) employment status (e.g., full-time, part-time, on-call, volunteer, contract); 4) whether the position is unionized; 5) annual salary; and 6) additional jobs held, for any reason. These questions will help gauge the individual and organizational qualities and characteristics that contribute to VSPs well-being.

#### Job satisfaction

The Profile Analysis of Job Satisfaction (PAJS-FI; 27) is an 11-item scale to assess an individual’s perception of being satisfied with their job. The items measure the following facets of job satisfaction: 1) information and communication; 2) organization and management; 3) working conditions; 4) relationships between the respondent and colleagues and direct supervisors; 5) demands of the job; 6) decision range; 7) working conditions; 8) working hours; and 9) vacation time, benefits, and compensation. Respondents are asked to rate their level of satisfaction with each of these facets using a 5-point Likert scale, ranging from 1 (very satisfied) to 5 (very unsatisfied). The values of the Likert scale are inverted so that high values were equal to high job satisfaction [27]. The PAJS-FI was chosen specifically for this study because job satisfaction is a key indicator of job performance and can help employers make informed decisions for the benefit of employees. Using facet items to measure job satisfaction is more efficient and cost-effective, but still gives accurate results when compared to using facet scales [27]; it is for these reasons that we selected this scale for inclusion in this study.

#### Turnover intention

A total of two items will be adapted to measure turnover intention [28]. These items are scored on a 5-point Likert-type scale that ranges from 1 (*Strongly disagree*) to 5 (*Strongly agree*). Higher scores correspond with greater endorsement of one’s intention to quit their current job and find new employment. We will include a question using the same Likert-scale asking participants if they worry about funding for their position or organization. Following this item, two additional questions (one “check all that apply” and the other open-ended) will be asked regarding the potential reasons a participant may be considering leaving their job.

#### Microaggressions

A total of six items will be used to determine the occurrence of microaggressions in the organization based on previous work [29]. These items capture whether microaggressions may have been experienced or witnessed by the participant. These items are scored as either ‘Yes,’ ‘No’ or ‘Don’t know.’ An open-ended question is included to explore what happened and how the issue was addressed, if known. These questions will help determine where microaggressions may be occurring, who is involved, and broad circumstances of the event.

#### PTSD symptomology

The Primary Care PTSD Screen for DSM-5 (PC-PTSD-5; 30) consists of five yes/no items and is used to identify individuals with probable PTSD. Each item is scored dichotomously with respondents indicating either the presence of *DSM-5* PTSD symptoms (score of 1) or the absence of a symptom (score of 0). Thus, the greater score an individual receives indicates probable *DSM-5* PTSD. The optimal cut-off score for probable PTSD is 3. Sample questions include, “Been constantly on guard, watchful, or easily startled?” and, “Felt numb or detached from people, activities, or your surroundings?” The scale has strong predictive validity (AUC = .94; 95% CI [.91, .97]; [30]) and good internal consistency (α = .87), test-retest reliability (*r* = .89), and concurrent validity (*r* = .81) [31].

#### Compassion fatigue and compassion satisfaction

The Professional Quality of Life Scale (ProQOL), Version 5 [32] is a validated 30-item self-report measure that consists of three subscales: compassion satisfaction, compassion fatigue, and burnout. The compassion fatigue subscale captures two parts: burnout and secondary traumatic stress. Items are scored on a 5-point Likert scale that ranges from 1 (*Never*) to 5 (*Very Often*) and are based on participants’ opinions within the last 30 days. For each subscale, scores below 22 are considered ‘Low,’ scores between 23–41 are considered ‘Average,’ and scores above 42 are considered ‘High.’ Thus, greater scores on each subscale indicate higher levels of the respective construct (e.g., a higher score on the compassion satisfaction subscale indicates higher levels of compassion satisfaction). The ProQOL subscales have acceptable reliability and validity (α = .88 for compassion satisfaction, α = .75 for burnout, and α = .81 for secondary traumatic stress); it also has good construct validity with over 200 papers published [32].

#### Vicarious resilience

The Vicarious Resilience Scale [VRS; 18] is a validated 27-item self-report measure consisting of seven factors: 1) increased capacity for resourcefulness; 2) client-inspired hope; 3) increased consciousness about power and privilege relative to clients’ social location; 4) changes in life goals and perspective; 5) increased self-awareness and self-care practices; 6) increased recognition of clients’ spirituality as a therapeutic resource; and 7) increased capacity for remaining present while listening to trauma narratives. Items are scored on a 6-point Likert-type scale with total scores ranging from 0 to 135. Higher scores indicate greater vicarious resilience. The internal consistency reliability for the overall VRS was very good (α = .94) and ranged from .77 to .86 for the seven subscales [18].

#### Coping strategies

The study will use a modified version of The Time Spent subscale in the Coping Strategies scale [33, 34]. The modified version is a 20-item self-report measure that assesses participant’s coping behaviours. The measure consists of three subscales: 1) the leisure subscale, which asks about frequency of time spent with family or hobbies; 2) the self-care subscale, which asks about self-care activities such as spending time with family and stress management activities; and 3) a subscale asking about advocacy activities such as research and political action regarding intimate partner violence and sexual assault that may serve as a coping mechanism. Items are scored on a 4-point Likert-type scale ranging from 1 (*Not at all*) to 4 (*Frequently*), with an additional option to choose ‘*Not applicable*.’ The measure has demonstrated acceptable psychometric properties with a good internal consistency (α = .87; 34). This scale was selected for the study because it has been used in past research with VSPs, has good psychometric properties, and will help determine how respondents are managing their overall well-being.

#### Open-ended questions

The final section of the survey will ask open-ended questions to gain a deeper understanding of participants’ propensity towards any personal changes, the impacts of COVID-19 on one’s wellness, and overall vicarious resilience (e.g., how has working or volunteering with victims or survivors of crime affected you personally?). These open-ended questions serve multiple purposes. First, it allows for the identification of any systemic or organizational barriers that may affect well-being. Second, it explores the growth or change that may occur from working as a VSP and identifies the coping tools currently utilized by VSPs.

### Plan for data analyses

The data analysis will involve both quantitative and qualitative approaches. The robustness of the data, the size of the sample, and the wide variability in the respondent target group will offer several avenues for potential themes to be investigated and relationships explored.

#### Quantitative analysis

Descriptive statistics (e.g., frequencies, percentages, central tendencies, etc.) will describe the sample and explore all demographic and organizational variables of interest. Some comparative analyses may be possible (e.g., government vs. non-government agencies, indeterminate vs. contract positions), as well as sector reports by organization type (e.g., shelter, sex assault centre, child protection, etc.) and region (e.g., Ontario, Atlantic provinces, etc.).

Inferential analyses will also be completed to identify the significance of differences between groups (e.g., ANOVA) on various demographic and organizational measures and survey scale data (e.g., PAJS-FI, VRS, ProQOL, PC-PTSD-5, etc.). Multiple linear regression models to identify predictor variables of compassion satisfaction, compassion fatigue, or secondary traumatic stress can be completed. Path analysis may be conducted to explore mediator effects related to job satisfaction and turnover intention. Also, the VRS could be further validated using factor analysis on the sample obtained. All quantitative analyses will be conducted using IBM SPSS version 28.

### Qualitative analysis

We will conduct a thematic analysis of the qualitative data. We will upload the open-ended survey questions, interviews, and focus group data into ATLAS.ti. We will use provisional coding to develop a list of a priori codes from our research questions [35]. We will incorporate emergent codes into the codebook during analysis [36]. A team of approximately five research assistants who meet at weekly lab meetings will complete the coding; this work will begin collaboratively before moving to more independent coding once interrater reliability reaches at least 75 percent [37]. To determine at what point we reached data saturation, we will have weekly team debriefs to discuss new and ongoing themes and continue our data collection until we stop identifying new themes. We will use the qualitative data to provide more context to the quantitative analyses.

## Discussion

A current, large-scale study of Canadian VSPs is needed to address a gap in literature and further understanding of the concept of vicarious resilience for those working in the trauma field. By identifying the factors that affect VSPs well-being, better support may be provided to them and the volunteers who provide trauma care to victims and survivors of crime in Canada. Peer-reviewed and open access articles, as well as the creation of sector-specific toolkits and training materials, will help disseminate these findings to practitioners and academics alike.

### Strengths and limitations

There are multiple strengths in this protocol. First, outreach to and partnership with sector professionals and the Federal-Provincial-Territorial (FPT) Working Group on Victims of Crime lends itself to a study that is practitioner-informed, thereby reducing the research-practice gap. Additionally, it increases the breadth and reach of recruitment efforts throughout Canada. Offering multiple modes of participation—survey, focus group, or semi-structured interview—is another strength; this not only increases potential participation and engagement, it also provides ample opportunity for participant voices to be heard. The mixed method design is also a strength. Not only can it provide quantitative data to make inferential comparisons, the qualitative data adds additional layers of complexity and nuance not often captured in mono-method research.

A potential limitation surrounds the question about organizational descriptions. The response option—‘mark all that apply’—may limit some comparison across sectors due to overlap in organizational mandates. An additional limitation may result from small sample sizes across some sectors or groups. Lastly, despite many sectors demonstrating managerial support for VSPs to complete the survey during working hours, it is possible that some interested participants are not able to devote their time to a survey of this length in a voluntary capacity.

### Knowledge dissemination

To inform and engage VSPs, our partner organizations, and academia, we plan to create the following resources:

1. Toolkits: Evidence-based research on well-being will be integrated with grounded examples and practices from our research with VSPs to develop a practical toolkit to support vicarious resilience. These tools will be disseminated through our outreach networks and made available for free online. A copy of the toolkit will be hosted on Victim Justice Network (VJN) and Victimology Research Centre (VRC) websites.
2. VSP Training: We will develop accessible and engaging training materials with our collaborators. Members of the research team will facilitate virtual and in-person training in both Canadian official languages.
3. Sector Reports: These brief reports will provide an overview of findings across different sectors. We will include information about what VSPs find most helpful in supporting survivors, sector-specific systemic barriers encountered, information on their own well-being, and additional training needs. These reports will build a powerful portrait of how victim services are operating across the country and can be shared through our partner networks and hosted on relevant websites (e.g., VJN, VRC).
4. New Partnerships: For example, plans are underway to partnership with Women’s Shelters Canada on the Feminist Brain Drain Study that will examine worker wellness and retention in violence against women (VAW) shelter workers. We will also prioritize international partnerships, such as working with the VRS developers and others with expertise in job satisfaction, burnout, and turnover.
5. Mobilization: We will disseminate findings at both local and international academic and practitioner-focused conferences (e.g., the Canadian Sociological Association, World Society of Victimology, etc.), as well as through various government reports (e.g., Research Digest with the Department of Justice Canada) and peer-reviewed journals (e.g., International Journal of Victimology).
