# Supplementary material for: Protocol for a study on vicarious resilience in service providers for victims and survivors of violence
Source: PLoS One. 2023 Mar 23;18(3):e0283474. doi: 10.1371/journal.pone.0283474 (PMC10035828; doi:10.1371/journal.pone.0283474)
Supplement: Peer review history

# Editor report: Decision Letter 0

**DOI:** 10.1371/journal.pone.0283474.r001

**Authors:** Avanti Dey

9 Mar 2023

Protocol for a Study on Vicarious Resilience in Service Providers of Victims and Survivors of Violence​

PONE-D-23-04664

Dear Dr. Ferns,

We’re pleased to inform you that your manuscript has been judged scientifically suitable for publication and will be formally accepted for publication once it meets all outstanding technical requirements.

Within one week, you’ll receive an e-mail detailing the required amendments. When these have been addressed, you’ll receive a formal acceptance letter and your manuscript will be scheduled for publication.

An invoice for payment will follow shortly after the formal acceptance. To ensure an efficient process, please log into Editorial Manager at http://www.editorialmanager.com/pone/, click the 'Update My Information' link at the top of the page, and double check that your user information is up-to-date. If you have any billing related questions, please contact our Author Billing department directly at authorbilling@plos.org.

If your institution or institutions have a press office, please notify them about your upcoming paper to help maximize its impact. If they’ll be preparing press materials, please inform our press team as soon as possible -- no later than 48 hours after receiving the formal acceptance. Your manuscript will remain under strict press embargo until 2 pm Eastern Time on the date of publication. For more information, please contact onepress@plos.org.

Kind regards,

Avanti Dey

Staff Editor

PLOS ONE

Journal Requirements:

1.  Your ethics statement should only appear in the Methods section of your manuscript. If your ethics statement is written in any section besides the Methods, please move it to the Methods section and delete it from any other section. 

Additional Editor Comments:

Thank you for submitting your work to PLOS ONE. Your submission has been reviewed to be eligible for an expedited decision, and you will receive details of any further information we need from you. Thank you again!

# Editor report: Acceptance letter

**DOI:** 10.1371/journal.pone.0283474.r002

**Authors:** Avanti Dey

15 Mar 2023

PONE-D-23-04664 

Protocol for a Study on Vicarious Resilience in Service Providers for Victims and Survivors of Violence 

Dear Dr. Ferns:

I'm pleased to inform you that your manuscript has been deemed suitable for publication in PLOS ONE. Congratulations! Your manuscript is now with our production department. 

If your institution or institutions have a press office, please let them know about your upcoming paper now to help maximize its impact. If they'll be preparing press materials, please inform our press team within the next 48 hours. Your manuscript will remain under strict press embargo until 2 pm Eastern Time on the date of publication. For more information please contact onepress@plos.org.

If we can help with anything else, please email us at plosone@plos.org. 

Thank you for submitting your work to PLOS ONE and supporting open access. 

Kind regards, 

PLOS ONE Editorial Office Staff

on behalf of

Dr. Avanti Dey 

Staff Editor

PLOS ONE
